# Optimization of Amylase Production from *B. amyloliquefaciens* (MTCC 1270) Using Solid State Fermentation

**DOI:** 10.1155/2014/764046

**Published:** 2014-05-11

**Authors:** Koel Saha, Sujan Maity, Sudeshna Roy, Koustav Pahan, Rishija Pathak, Susmita Majumdar, Suvroma Gupta

**Affiliations:** Department of Biotechnology, Haldia Institute of Technology, ICARE Complex, Purba Medinipur, West Bengal 721657, India

## Abstract

Demand for microbial amylase production persists because of its immense importance in wide spectrum industries. The present work has been initiated with a goal of optimization of solid state fermentation condition for amylase using agroindustrial waste and microbial strain like *B. amyloliquefaciens* (MTCC 1270). In an aim to improve the productivity of amylase, fermentation has been carried out in the presence of calcium (Ca^+2^), Nitrate (NO_3_^−^), and chloride ions (Cl^−^) as well as in the presence of D-inositol and mannitol. Amylase needs calcium ion for the preservation of its structure, activity and stability that proves beneficial also for amylase production using solid state fermentation. The inclusion of ions and sugars in the SSF media is promising which can be explained by the protection offered by them against thermal decay of amylase at various incubation periods at 37°C.

## 1. Introduction

Alpha-amylase [EC 3.2.1.1] cleaves the 1,4-*α*-D-glycosidic linkages between adjacent glucose unit inside the linear starches, glycogen, and oligosaccharides in a random manner [1]. Multifarious uses of alpha-amylases as a major starch degrading agent in food, paper, textile, and brewing industry necessitates its prolific production that can be effectively met up by solid state fermentation (SSF) [2]. Agrowastes like wheat bran, rice bran, and coconut oil bran have replaced the high cost media generally used in submerged fermentation for alpha-amylase preparation because of their simplicity, low cost, easy availability, better productivity, and lesser water output. Additionally it solves the pollution problem occurring due to their disposal in the surrounding [3]. High starch content of almost all agrowastes (60–70% by weight) can be effectively utilized as a major nutrient source by microorganisms like bacteria, fungi, and so forth, for the synthesis of inducible alpha-amylase which is under the control of catabolic repression.

Plethora of evidences exists in favor of wheat bran as the best sources among all the agrosources for extracellular amylase production [4, 5]. Based on the prior knowledge of primary solid state fermentation culture condition, the present study was initiated using wheat bran as a prime source of nutrient and* B. amyloliquefaciens *(MTCC 1270) as the producer organism at pH 7 to increase the alpha-amylase yield through media optimization.Earlier reports are also in agreement with the fact that most of the* Bacillus* species, namely,* Bacillus licheniformis* and* Bacillus stearothermophilus*, are the most effective producers of alpha-amylase [5–11].

Most of the amylases are metalloenzyme requiring Ca^+2^ for their activity, structural integrity, and stabilization [12–14]. At least three calcium binding sites have been located on barley alpha-amylase isoform that is also visible for plants, mammals, fungi, and bacteria [15, 16]. For* B. amyloliquefaciens*, the calcium binding site is contributed by three conserved regions of the polypeptide chain comprising residues Gly^97^-Ala^109^, Ile^217^-His^235^, and Ser^314^-Ser^334^ [17]. Depletion of calcium ion from the binding site abolishes amylase activity. Similar stabilization effect has been provided by chloride and nitrate ions as reported by Aghajari et al. [18]. In this work major emphasishas been given in search of conditions as well as for parameters like ions and sugar alcohols whose presence in the fermentation media stimulates alpha-amylase production from SSF.

## 2. Materials and Methods

### 2.1. Microorganism

*Bacillus amyloliquefaciens *(MTCC 1207, IMTECH, Chandigarh) was used as working strain for solid state fermentation (SSF) extraction of alpha-amylases. All the reagents are of analytical grade (SRL).

### 2.2. Preparation of Inoculum and Solid State Fermentation (SSF)

Wheat bran was collected from local market and solid state fermentation has been carried out with 4 gm dry wheat bran in a 100 mL Erlenmeyer flask. The moisture level of the wheat bran was adjusted to 50% (w/w) with autoclaved distilled water. The contents of the flask were autoclaved prior to the solid state fermentation.

25 mL of nutrient broth was taken in a 100 mL flask and was inoculated with a loop full of* Bacillus amyloliquefaciens* cells from a 24-hour-old slant and kept at 37°C in a shaker. After 16 hours of growth, 1 mL inoculum (1.5–2 × 10^8^ cfu/mL) from this broth culture was added in the WB. It was fermented for various fermentation periods (24 and 48 hours) at different temperatures (30°, 33°, 37°, and 42°C).

### 2.3. Enzyme Extraction

After 24 and 48 hours of fermentation, the fermented media containing wheat bran were mixed with 25 mL 20 mM phosphate buffer (pH = 7.0) for 30 minutes at 4°C in a rotary shaker at 150 rpm. The suspension was then centrifuged at 8000 rpm for 15 min at 4°C. The supernatant has been collected and used for amylase assay.

### 2.4. Amylase Assay

Alpha-amylase activity of the extract was measuredby DNS method [19]. In briefthe reaction mixture containing 1% soluble starch, 20 mM phosphate buffer (pH = 7), and fermented extract was taken and incubated at 37°C for 20 minutes followed by the addition of 3,5-dinitrosalicylic acid (DNS). The amount of the reducing sugar liberated during assay was estimated by measuring color development at 540 nm by UV-VIS spectrophotometer. 1U of amylase activity is defined as the amount of enzyme that liberated micromole of maltose per minute under standard assay condition.

### 2.5. Protein Estimation

The protein content of the extract was determined following Lowry's method [20].

### 2.6. Starch Hydrolysis

A 2% starch agar plate (beef extract—0.3%, soluble starch—1%, and agar—2%) has been prepared and streaked from a 24-hour-old culture of* Bacillus amyloliquefaciens*. The plate was grown for 48 hours in 37°C. To check the starch hydrolysis property of alpha-amylase the plate was flooded with iodine solution.

### 2.7. Optimization of Media

4 gram of WB was supplemented with various concentrations of ions like Ca^+2^, Cl^−^, and NO_3_^−^ (0.1, 0.2, and 0.4 M) from 0.5 M respective stocks of CaCl_2_, NaCl, and NaNO_3_ salt solutions for a comparative analysis regarding the yield of alpha-amylase with that of the control WB. The relative humidity was kept constant at a level of 50% (w/w) with autoclaved distilled water. The content of the flask was autoclaved and tested for solid state fermentation for 48 hours at 37°C with the addition of 1 mL inoculum (1.5–2 × 10^8^ cfu/mL) from the broth culture. The extraction of the enzyme was performed following the same procedure as described earlier. Similar protocol of SSF has been followed for 0.5 and 1% D-inositol and D-mannitol supplementation into the WB, with proper moisture level adjusted. Control WB was autoclaved and kept for solid state fermentation under similar experimental condition without any salt and sugar supplementation with equal inoculums size as earlier. The alpha-amylase activity has been calculated according to DNS method [19].

### 2.8. Statistical Analysis

Effect of each parameter was studied in triplicate and graphically represented as the mean ± SD (*n* = 3) using Origin 5.

## 3. Results

### 3.1. Amylase Is Able to Hydrolyze Starch

**Figure 1:**
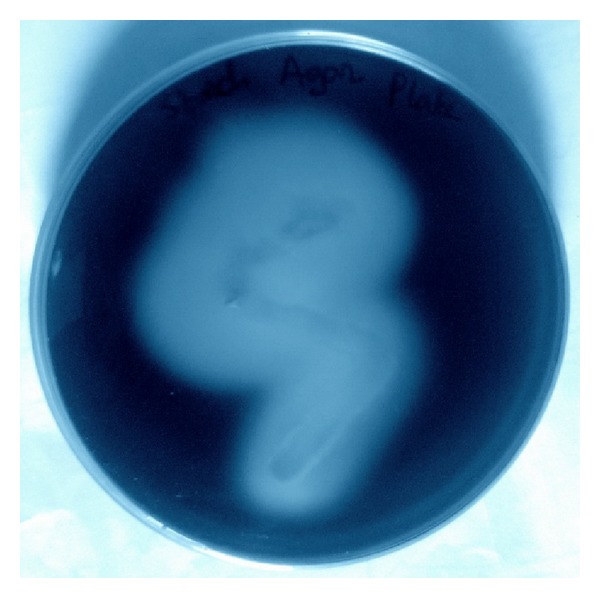
Starch hydrolysis performed on a 2% starch agar plate using* B. amyloliquefaciens *(MTCC 1270).

The starch agar plate was inoculated with* B. amyloliquefaciens* (MTCC 1270) and kept for 48 hours at 37°C. The plate was flooded with iodine and clear zone of starch hydrolysis has been observed (Figure 1). This ensures that this microorganism secretes amylase that is capable of starch hydrolysis.

### 3.2. Production of Alpha-Amylase from* B. amyloliquefaciens* (MTCC 1270) Using Solid State Fermentation

**Figure 2:**
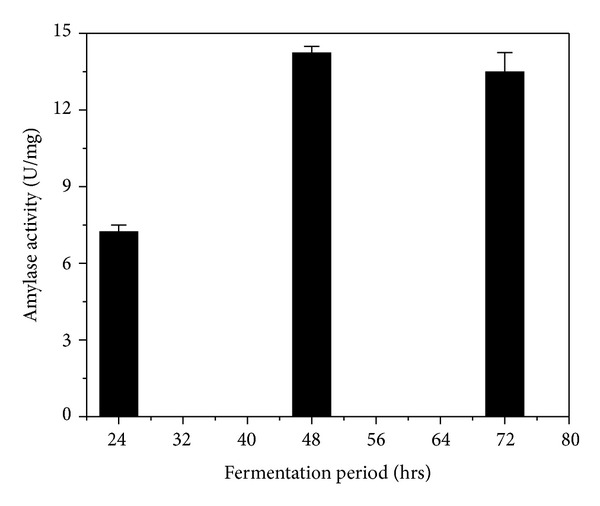
*α*-Amylase production from solid state fermentation using* B. amyloliquefaciens *(MTCC 1270) and wheat bran; black column: specific activity of *α*-amylase from SSF extract during different fermentation time periods.

To optimize the appropriate fermentation period for high yield alpha-amylase production, the study had been initiated with wheat bran and* B. amyloliquefaciens *(MTCC 1270) for 24, 48, and 72 hours. The values of specific activity of alpha-amylase were 7.25 ± 0.25 U/mg, 14.25 ± 0.24 U/mg, and 13.5 ± 0.75 U/mg, respectively, after 24, 48, and 72 hours using SSF under identical fermentation conditions (time and temperature) (Figure 2). Fermentation conducted for longer period of time was accompanied with decline in the alpha-amylase activity caused by denaturation and degradation of enzyme products.

### 3.3. Influence of Temperature on Amylase Production from SSF

Temperature had profound effect on the growth of the microorganism as well as on the enzyme activity.

**Figure 3:**
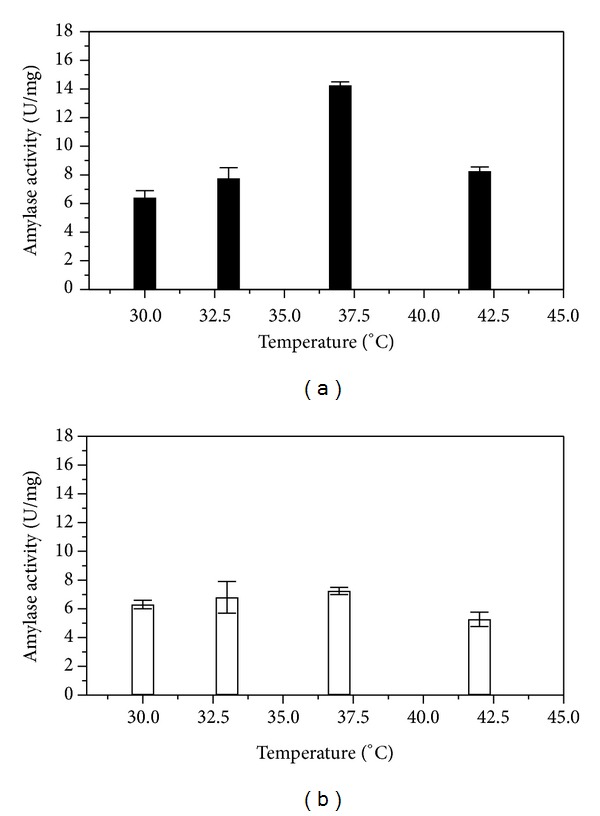
Effect of temperature on *α*-amylase yield after various periods of fermentation using* B. amyloliquefaciens* (MTCC 1270); white column: specific activity of *α*-amylase from 24 hours SSF extract; black column: specific activity of *α*-amylase from 48 hours SSF extract.

Effect of temperature on alpha-amylase production through solid state fermentation had been tested for two fermentation hours (24, 48) and at four different temperatures (30°, 33°, 37°, and 42°C). A 24-hour SSF at 37°C yielded maximum alpha-amylase production with an activity (7.25 ± 0.25 U/mg) that had been further enhanced with longer fermentation period after 48 hours at the same temperature. Although alpha-amylase production was evident at all the four temperatures studied for fermentation, 37°C was the best among all to produce maximum amylase from SSF with a specific activity of 14.25 ± 0.24 U/mg (Figure 3). This result corroborated well with optimum temperature of alpha-amylase (data not shown) that came around 40°C using standard DNS assay method [19]. After 42°C alpha-amylase activity declined due to the metabolic heat generated as an outcome of microbial growth in the solid state fermentation medium.

### 3.4. Effect of Ions Present in the SSF Media on Alpha-Amylase Production

**Figure 4:**
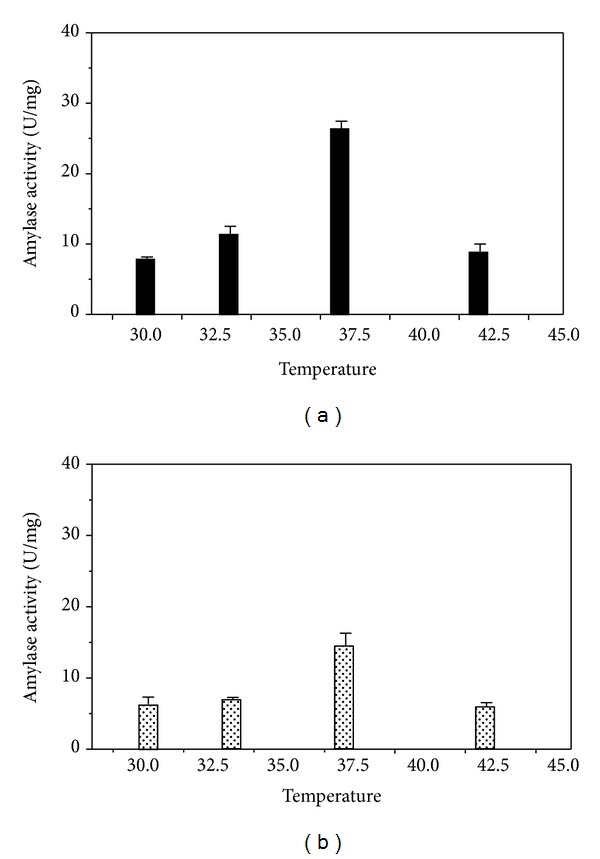
Effect of 0.1 mM calcium (Ca^+2^) on amylase production after 48 hours of SSF at 37°C temperature; dotted column: specific activity of *α*-amylase in absence of calcium ion from SSF extract; black column: specific activity of *α*-amylase in presence of calcium ion from SSF extract.

Effect of calcium (Ca^+2^) on amylase production through solid state fermentation had been checked for 48 hours fermentation at four different temperatures (30, 33, 37, and 42°C). Effect of Ca^+2^ at a concentration of 100 mM had been tested with a control (without any ion). Compared to control the yield of alpha-amylase increased in presence of Ca^+2^ (Figure 4). Among all the temperatures, 37°C solid state fermentation carried out with calcium ion gave maximum alpha-amylase activity (27 ± 1.05 U/mg) where as in absence of calcium it was about 50% less (15 ± 1.75 U/mg). This indicated the supportive role of calcium (Ca^+2^) in the preservation of amylase structural integrity and stability [15]. There was a gradual increase in the specific activity of amylase from 30°C to 37°C in presence of calcium (Ca^+2^) with a downfall of amylase activity at 42°C (9.5 ± 1.1 U/mg).

**Figure 5:**
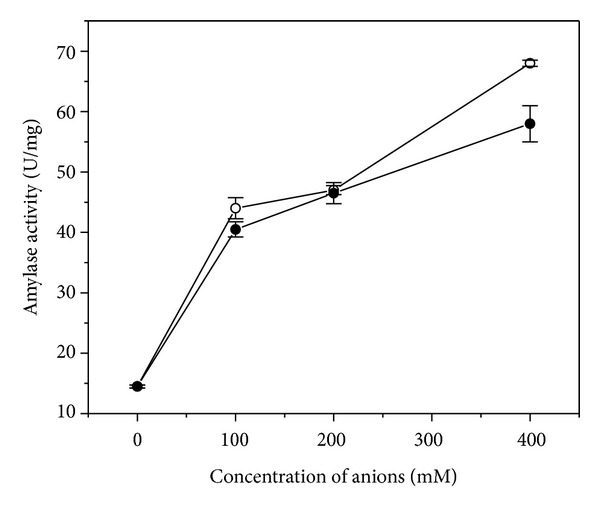
Effect of anions (nitrate and chloride ions) on amylase production after 48 hours of SSF, (○): specific activity of *α*-amylase in presence of nitrate ion from SSF extract; (●): specific activity of *α*-amylase in presence of chloride ion from SSF extract.

Effect of chloride and nitrate ion at various concentration ranges (100, 200, and 400 mM) had been tested in order to check the effect of negative ions on the alpha-amylase yield from SSF with a control (without any ion). The result was noteworthy with respect to improved amylase activity in presence of both Cl^−^ and NO_3_^−^ salts in the SSF media. Presence of 400 mM chloride (Cl^−^) and (NO_3_^−^) in the fermentation mixture improved amylase yield from 14.5 ± 0.25 U/mg to 58 ± 3 U/mg and 68 ± 0.25 U/mg, respectively, compared to control without any salt (Figure 5). This observation can be correlated well with an insight to the alpha-amylase crystal structure derived from porcine pancreatic source at 5 Å resolutions. Chloride ion stabilized amylase structure by making electrostatic interaction with the neighboring positively charged residues like Arg 195, Lys 257, and Arg 337, which were on the other hand very close to the active site cleft of amylase. This was in congruence with the observation by Lifshitz and Levitzky, identifying one lysine residue close to the active site region that bonded with the chloride ion if present in the vicinity of the enzyme [21].

### 3.5. Influence of Supplementation of Sugar Alcohol on Amylase Production from SSF

**Figure 6:**
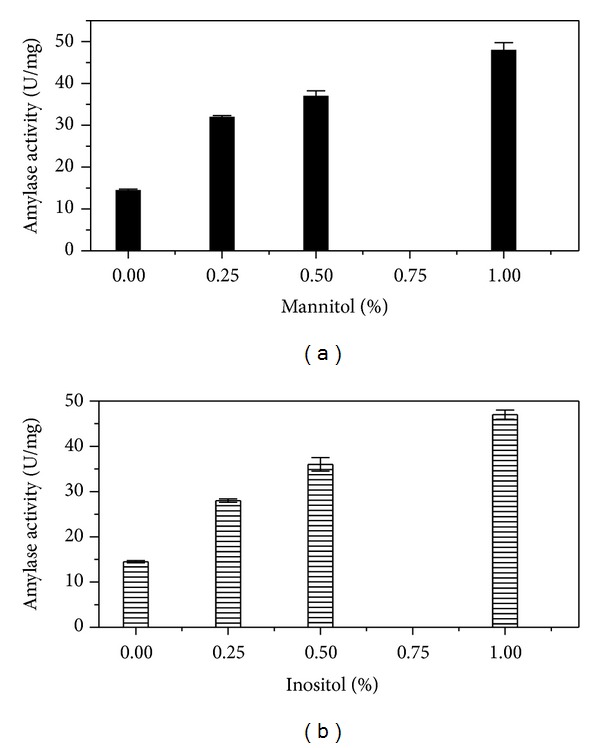
Effect of sugar alcohol (D-mannitol and D-inositol) on amylase production after 48 hours of SSF; lined column: specific activity of *α*-amylase in presence of inositol from SSF extract; black column: specific activity of *α*-amylase in presence of mannitol from SSF extract.

Being an inducible enzyme, alpha-amylase was sensitive to catabolite repression [22]. Addition of soluble starch encouraged amylase production by* B. amyloliquefaciens* [23]. SSF was conducted in presence and absence of D-inositol and mannitol at 37°C for 48 hours and the alpha-amylase activity had been presented in Figure 5. The increase in inositol and mannitol concentration in the fermentation media was accompanied with the rise in amylase activity (Figure 6). 1% inositol and mannitol had maximum amylase activity of 48.5 ± 1 U/mg and 51.24 ± 1.75 U/mg, respectively, compared to control 14.5 ± 0.25 U/mg.

**Figure 7:**
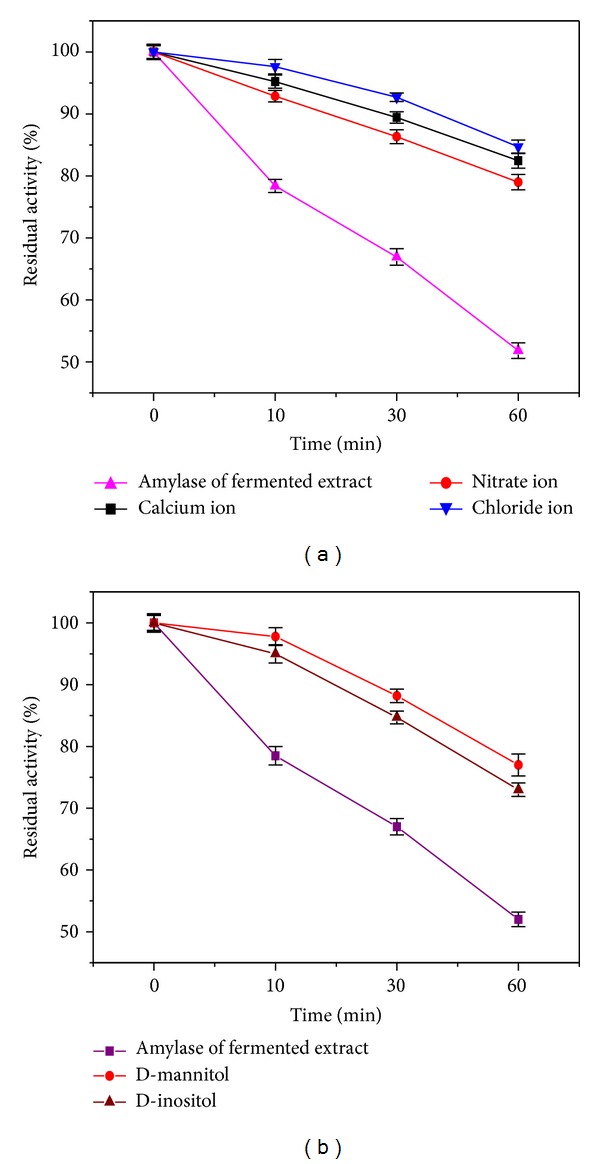
Percentage residual activity of amylase in absence and presence of ions and sugar alcohols.

In order to elucidate the role of all the supplements in enhancing alpha-amylase activity in the fermented extract, the extract containing alpha-amylase was subjected to thermal decay at 37°C temperature for various incubation periods ranging from 0 to 60 minutes in absence and presence of ions and sugar alcohols. D-Inositol and D-mannitol have offered considerable protection against heat induced denaturation at 37°C after one hour as manifested from the retention of residual enzyme activity around 73 and 77% compared to 52% observed for amylase in extract alone in absence of any stabilizer. Similar trend of stabilization of alpha-amylase activity in presence of various salt ions (100 mM) like calcium, chloride, and nitrate has also been noticed to be subjected under thermal denaturation under similar conditions as before. All the salt ions have protected around 80% of amylase activity compared to control without salts having activity around 52% (Figure 7).

## 4. Discussion

Solid state fermentation carried out with cheap source like wheat bran seemed to be promising for amylase production using* Bacillus amyloliquefaciens*. Optimization of different fermentation hours and temperatures for solid state fermentation had been attempted and 48 hours solid state fermentation at 37°C gave maximum amylase yield with wheat bran as major nutrient source. This was in agreement with the earlier reports by number of workers that elicited solid state fermented production of alpha-amylase at the range of temperatures from 37–60°C using number of* Bacillus* species [24–27]. A wide range of temperature from 35–80°C had also been proved effective for amylase production using various bacterial species [28–30]. With an aim to improve the amylase yield, solid state fermentation had been conducted with different ion (chloride, nitrate, and calcium) fortifications. The yield of alpha-amylase had been significantly improved in the fermentation mixture in presence of ions. Sugar alcohol supplementation of D-inositol and D-mannitol supported alpha-amylase production as manifested from the increase in yield of alpha-amylase in the fermentation media. Role of calcium as well as chloride ion in stabilization of amylase structure had been reported earlier by many workers [31]. Role of calcium ion in the stability and catalytic activity of alpha-amylase had been a topic of research since years. Presence of calcium ion in the fermentation media was stimulatory as it increased the yield of amylase from 15 U/mg to 27 U/mg in presence of 100 mM Ca^+2^. This corroborated well with earlier reports discerning the ability of Ca^+2^ to enhance amylase stability and activity from* Bacillus* spp [10, 32, 33]. Ca^+2^ significantly improved amylase production by* B. sphaericus* and* B. amyloliquefaciens* from SSF [34, 35]. Equally revealing was the information that addition of Ca^+2^ in the media accelerated amylase production by* Bacillus* spp. as observed by a number of workers [36–40]. It can been predicted from the crystal structure of amylase that calcium ion was involved in ionic interaction with charged residues like Asn 100, His 201 of domain A, and Asp 159 and Asp 167 of domain B of amylase. Active site of amylase was located between domains A and B and calcium ion formed an ionic bridge between A and B domains of amylase promoting its stability and catalytic activity [21].

Allosteric activation of amylase by chloride ion has been reported by D'Amico et al. in some Gram negative bacteria such as* Pseudoalteromonas haloplanktis* [30, 41]. NO_3_^−^ or ClO_3_^−^ also strengthened amylase activity delineating the fact that any negative charge played a pivotal role facilitating starch degradation reaction [18, 42, 43]. Compared to chloride, nitrate had offered better stabilization to amylase owing to its planer, triangular geometry that could penetrate well to the active site of alpha-amylase. An insight to the crystal structure of PPA at 5 Å resolutions as discussed earlier was in agreement with the supportive role of chloride ion as stabilizer. This was in conformity with the present observation that presence of negative ions in the production media as well as in assay mixture enhanced amylase activity (data not shown).

Inclusion of sugar alcohol like D-inositol and D-mannitol in the SSF production media improved amylase yield by 3.5-fold with respect to the control. Result was consistent with the findings by Srivastava and Baruah (1986) [44] that also supported improved alpha-amylase production using D-inositol in the SSF media [17, 18]. Ions and sugar alcohol might be protecting alpha-amylase against heat induced denaturation by offering stabilization through hydrogen bond formation with polar residues of amylase due to the presence of number of hydroxyl groups on D-inositol and D-mannitol.

In conclusion, amylase production using supplementation of ions and sugars in the solid state fermentation media seemed to increase the yield of amylase that can be propagated in SSF which is carried out with other* Bacillus* species. However this study delineated the supportive role of stabilizing ions and sugars to improve the amylase yield from SSF and can be useful as digestive because of its starch liquefaction property.
